# Symptom Duration, Recurrence, and Long-Term Effects of Swimming-Induced Pulmonary Edema: A 30-Month Follow-Up Study

**DOI:** 10.1016/j.chest.2023.06.041

**Published:** 2023-07-05

**Authors:** Linda Kristiansson, Claudia Seiler, Daniel Lundeqvist, Annika Braman Eriksson, Josefin Sundh, Maria Hårdstedt

**Affiliations:** a Center for Research and Development, Uppsala University/Region Gävleborg, Gävle, Sweden; b School of Medical Sciences, Faculty of Medicine and Health, Örebro University, Örebro, Sweden; c Sandviken North Primary Health Care Center, Sandviken, Sweden; d Center for Clinical Research, Dalarna-Uppsala University, Falun, Sweden; e Department of Anesthesiology and Intensive Care, Falun Hospital, Falun, Sweden; f Department of Internal Medicine, Mora Hospital, Mora, Sweden; g Vansbro Primary Health Care Center, Vansbro, Sweden; h Department of Respiratory Medicine, Faculty of Medicine and Health, Örebro University, Örebro, Sweden

**Keywords:** cohort, exercise, long-term effects, recurrence, SIPE, symptom duration, swimming-induced pulmonary edema

## Abstract

**Background:**

Swimming-induced pulmonary edema (SIPE) has been reported to subside within 24 to 48 h, but comprehensive follow-up studies on symptom duration and long-term effects are missing.

**Research Question:**

What are the symptom duration, recurrence, and long-term effects of SIPE?

**Study Design and Methods:**

A follow-up study was conducted, based on 165 cases of SIPE from Sweden’s largest open-water swimming event with 26,125 individuals participating during 2017-2019. Data on patient characteristics, clinical findings, and symptoms were collected at admission. Telephone interviews at 10 days and 30 months were performed to explore symptom duration, recurrence of SIPE symptoms, need for medical evaluation, and long-term effects of self-assessed general health and physical activity level.

**Results:**

Follow-up at 10 days was performed for 132 cases and at 30 months for 152 cases. Most of the patients were women, and their mean age was 48 years. At the 10-day follow-up, symptom duration > 2 days after the swimming race was reported by 38%. The most common symptoms were dyspnea and cough. In patients at 30-month follow-up, recurrence of respiratory symptoms during open-water swimming was reported by 28%. In multivariable logistic regression, asthma was independently associated with both symptom duration > 2 days and recurrence of SIPE symptoms (*P* = .045 and *P* = .022, respectively). Most participants reported equal or improved general health (93%) and physical activity level (85%) after experiencing SIPE, but 58% had not swum in open water since the event.

**Interpretation:**

The present large cohort study challenges the established hallmark of SIPE symptom duration < 48 h, whereas SIPE recurrence was in the previously reported range. At 30 months, most patients reported unchanged self-assessed general health and physical activity level. These findings add to our understanding of the course of SIPE and can provide evidence-based information to swimmers and health care professionals.

FOR EDITORIAL COMMENT, SEE PAGE 1077

**Take-Home Points**

**Study Question:** What are the symptom duration, recurrence, and long-term effects of SIPE?

**Results:** In total, 38% of patients with SIPE reported symptom duration > 2 days and 28% reported recurrence of SIPE symptoms, whereas self-assessed general health and physical activity level were not affected up to 30 months.

**Interpretation:** The present large cohort study challenges the established hallmark that SIPE symptoms subside within 48 h, whereas the recurrence of SIPE fell within the previously reported range.

Swimming-induced pulmonary edema (SIPE) was first described in 1989 and is characterized by cough and dyspnea, sometimes accompanied by frothy sputa and hemoptysis while swimming in open water.^1^ SIPE is a hydrostatic pulmonary edema hypothetically caused by central pooling of blood and peripheral vasoconstriction during immersion, in combination with strenuous exercise, while swimming in open water.^2^, ^3^, ^4^ The incidence of SIPE varies widely in the published literature (0.01%-26.7%), based on different criteria for SIPE diagnosis, study design, swimming conditions, and differences between study populations.^5^, ^6^, ^7^, ^8^, ^9^, ^10^ In the largest cohort study conducted so far, we reported an incidence of 0.44% in a heterogeneous population participating in the *Vansbrosimningen* open water swimming event in Sweden.^11^

SIPE is a condition that may be life-threatening, primarily because of the danger of drowning. Among divers experiencing a similar condition usually referred to as immersion pulmonary edema, occasional deaths have occurred.^12^ However, with timely interruption of swimming and exit from the water, SIPE has been reported to spontaneously subside within 24 to 48 h.^13^ The risk of SIPE recurrence varies in the literature (9%-40%) depending on how SIPE is defined, how SIPE incidence is estimated, and length of follow-up time.^7^, ^8^, ^9^, ^10^^,^^13^^,^^14^ The course, individual risk factors, comorbidity, and long-term effects of SIPE are mostly unknown, and comprehensive follow-up studies are lacking. To improve safety during open water swimming and to be able to provide swimmers with evidence-based individual guidance, a better understanding of the course and long-term effects of SIPE is needed.

The aim of this study was to explore symptom duration, recurrence, need for medical evaluation, and long-term effects after experiencing SIPE. Our study was based on follow-up data from a large heterogeneous cohort of patients with SIPE during *Vansbrosimningen*, Sweden’s largest open water swimming event.

## Study Design and Methods

### Settings and Study Population

*Vansbrosimningen* is the largest open water swimming event in Sweden, with about 11,000 individuals participating every year. The event takes place during a 3-day weekend in July, and consists of swimming distances of 1,000, 1,500, and 3,000 m in cold (15-20 °C) open water rivers. Available health care during the event consists of a first aid team along the riverside and a mobile medical unit (MMU) near the finish area. At the MMU, all swimmers in need of medical care are examined. All swimmers (≥ 18 years) participating in the 2017-2019 events who sought medical attention for cough and/or dyspnea, with acute onset during or immediately after the race, were eligible for the study.^11^^,^^15^ In the present study, all patients with SIPE were invited to follow-up interviews after 10 (± 2) days and 30 (± 5) months. Ethical approval was received from the regional ethical review board in Uppsala, Sweden (Dnr 2017/216, 2017/216/1, 2017/216/2, 2020-02265). Written informed consent was obtained from all participants.

### Criteria for Identifying Cases of SIPE

Diagnostic criteria for SIPE at *Vansbrosimningen* have been evaluated and refined over the years of the study. During the entire study, lung auscultation findings of crackles and peripheral oxygen saturation were noted for all patients with acute respiratory symptoms. In 2018 and 2019, lung ultrasound (LUS) was used to confirm pulmonary edema. Physicians on site were blinded to LUS results to evaluate the association of clinical findings related to objective signs of pulmonary edema.^15^ SIPE diagnosis at the MMU in 2017 and 2018 was based on findings of crackles during lung auscultation, and/or a peripheral oxygen saturation ≤ 95%. In 2019, we decided to use LUS findings for on-site diagnosis.

**e-Figure 1:**
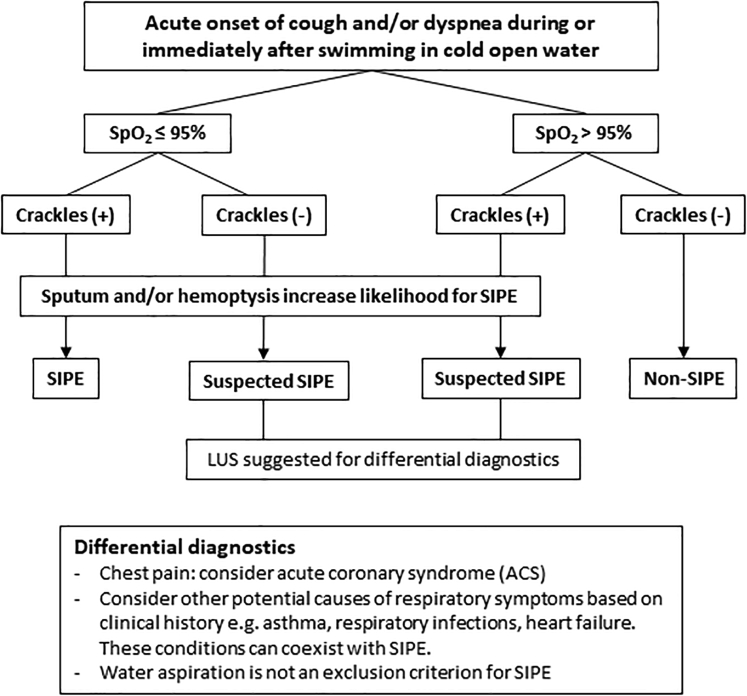


Data from 2018 and 2019 were assessed to create a diagnostic algorithm for SIPE (e-Fig 1).^15^ In the absence of LUS, this algorithm establishes SIPE on the basis of a combination of crackles during lung auscultation and/or peripheral oxygen saturation ≤ 95%.

**Figure 1:**
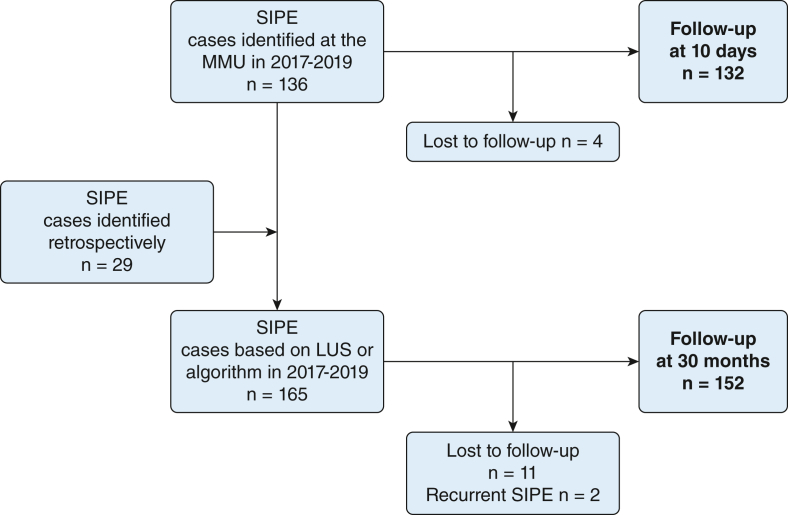
Study flow chart for follow-up interviews of patients with SIPE from Vansbrosimningen in 2017-2019. The 10-day follow-up was conducted for patients with SIPE identified on site at the MMU (n = 132). After standardization of SIPE diagnosis, 165 cases of SIPE were identified and available for the 30-month follow-up. Two patients sought medical attention with recurrent SIPE in 2 consecutive years and were contacted only once for the 30-month follow-up. LUS = lung ultrasound; MMU = mobile medical unit; SIPE = swimming-induced pulmonary edema.

In the present study, a 10-day follow-up was conducted on the basis of SIPE diagnosis at the MMU (Table 1). Before the 30-month follow-up, we reevaluated SIPE diagnosis for all patients to standardize diagnostics. SIPE diagnosis was based on LUS findings of pulmonary edema, or, in the absence of LUS data, the clinical algorithm was applied (e-Fig 1). This strategy identified 29 additional SIPE cases, the majority of which were patients from 2017 who appeared without crackles but with peripheral oxygen saturation ≤ 95% (n = 21) (Fig 1).

**Table 1:** Diagnostic Criteria for SIPE

Year	10-Day Follow-Up Clinical SIPE Diagnosis at MMU	30-Month Follow-Up Standardized SIPE Diagnosis
2017	Diagnosis based on lung auscultation and saturation (n = 38)^a^	Diagnostic algorithm (n = 59)^b^
2018	Diagnosis based on lung auscultation and saturation (n = 42)^a^	Pulmonary edema on LUS (n = 46); diagnostic algorithm (n = 1)^b^
2019	Pulmonary edema on LUS (n = 55) Diagnosis based on lung auscultation and saturation (n = 1)^c^	Pulmonary edema on LUS (n = 55); diagnostic algorithm (n = 4)^b^

LUS = lung ultrasound; MMU = mobile medical unit; SIPE = swimming-induced pulmonary edema.

a Clinical criteria for SIPE at the MMU in 2017 and 2018: crackles on lung auscultation and peripheral oxygen saturation ≤ 95% (n = 69), crackles and saturation > 95% (n = 6), crackles and missing saturation (n = 2), rhonchi and saturation ≤ 95% (n = 1), rhonchi and missing saturation (n = 1), missing lung auscultation and saturation ≤ 95% (n = 1).

b Algorithm presented by Hårdstedt et al.^15^

c LUS data were missing.

### Data Collection

Data on patient characteristics, clinical findings, and symptoms were collected at the MMU.^11^ Information on swimming experience in open water the same year as the swimming race and previous episodes of dyspnea and/or cough while swimming in open water was noted. Patients who required medical attention following the MMU visit or during follow-up were asked to provide their medical records. Records were assessed regarding hospital admissions, investigations, new diagnoses, and treatment. Structured telephone interviews were conducted at 10 days and 30 months by one of the four coauthors (L. K., C. S., D. L., and M. H.). Participants not reached by phone after three attempts received the questionnaires by mail (a total of 21 patients).

The questionnaires used during the interviews were developed for this purpose by our research group. Most of the questions were structured as “multiple choice” (e-Fig 2). A group consisting of swimmers who had previously experienced SIPE and fellow researchers evaluated the questionnaire for relevance and simplicity. Questions on general health and physical activity level were inspired and modified from the 36-Item Short-Form Survey (SF-36) and a validated form for skydivers.^16^^,^^17^ At both follow-ups, the questionnaires contained items on continuing symptoms, symptom duration, experiences of respiratory symptoms while swimming in open water after SIPE, need for medical evaluation after SIPE, and frequency of physical activities (endurance, strength, and swimming exercise) over the previous 12 months. Symptom duration in the questionnaire was categorized as < 1 day, 1 to 2 days, 3 to 4 days, and ≥ 5 days at the 10-day follow-up; and periods of 0 to 10 days, > 10 days to 1 month, > 1 to < 12 months, and ≥ 12 months at the 30-month follow-up. For 2018 and 2019, a question was added at 10 days about the frequency of swimming the year before the race.

In addition, the 30-month follow-up included questions about medical diagnoses obtained after the race. Two questions explored whether experiencing SIPE had affected their subjective general health status and physical activity level. Self-assessed general health status and physical activity level were also scored on a five-point scale and presented on a three-point scale.

### Data Analysis

The various cohorts used to evaluate outcome measures are summarized in e-Table 1. For baseline characteristics, continuous data are presented as means with SD, or as medians with interquartile ranges, and categorical data are presented as numbers with percentages. The proportion of patients with recurring SIPE symptoms during open water swimming was calculated, as was the proportion of patients with SIPE symptoms at open water swimming either before or after the race. Univariable and multivariable logistic regression analyses were conducted for the outcomes of symptom duration > 2 days and recurrence of SIPE*.* The two main models included age, and comorbid conditions in terms of hypertension and asthma at baseline as explanation variables. The McNemar test was used to compare patients’ answers for frequency of physical activity “never” and “ever” the year before the swimming race with the year before the 30-month follow-up. SPSS Statistics for Windows version 28.0 (IBM) and Prism version 9.4.1 (GraphPad Software) were used for statistical analysis and graphics presentation. A *P* value < .05 was considered statistically significant.

## Results

In total, 26,125 unique individuals (≥ 18 years; 53% women) participated in *Vansbrosimningen* in 2017-2019. Altogether, 246 patients were assessed at the MMU for respiratory symptoms and of these, 165 received a diagnosis of SIPE.

The 10-day follow-up was conducted for 132 SIPE cases and the 30-month follow-up for 152 cases (Fig 1, Table 1). Two of the patients received a diagnosis of SIPE in two different years. One of these patients was interviewed at 10 days in both years, the other patient only in 1 year. Both patients were interviewed only once at 30 months.

The vast majority of patients with SIPE were women and those who did not smoke; the most frequently reported comorbid conditions at the time of the swimming race were asthma and hypertension (Table 2).

**Table 2:** Characteristics of SIPE Cases Reported at MMU in 2017-2019

Characteristic	10-Day Follow-Up (n = 132)	30-Month Follow-Up (n = 152)	Total (N = 165)
Patient characteristics			
Age, mean (SD), y	48 (10)	48 (10)	48 (10)
Sex, female	121 (92)	136 (89)	149 (90)
BMI, median (IQR), kg/m^2^	23 (22-27)^a^	23 (22-27)^b^	24 (22-26)^c^
Smokes	1 (1)	2 (1)	2 (1)
Asthma	23 (17)	26 (17)	29 (18)
Hypertension	14 (11)	16 (11)	17 (10)
Heart disease	5 (4)	6 (4)	6 (4)
Symptoms at admission			
Dyspnea	107 (81)	124 (82)	133 (81)
Cough	107 (81)	125 (82)	135 (82)
Increased sputum	46 (35)	47 (31)	50 (30)
Hemoptysis	9 (7)	9 (6)	9 (6)
Clinical findings			
Oxygen saturation at admission, median (IQR)	91 (88-94)^a^	92 (89-95)^b^	92 (89-95)^b^
Crackles	124 (94)	122 (80)^d^	131 (79)^d^
Treatment			
CPAP	93 (71)	91 (60)	95 (58)
PEP device	24 (18)	23 (15)	24 (15)
Inhalation of β-agonist	10 (8)^b^	13 (9)^b^	15 (9)^b^
Furosemide	2 (2)^b^	2 (1)^b^	2 (1)^b^
Swimming experience and earlier symptoms			
Open-water swimming before the race^e^			
Never	32 (24)	36 (24)	42 (26)
1 or 2 times	37 (28)	46 (30)	47 (29)
3-5 times	36 (27)	40 (26)	43 (26)
> 5 times	27 (21)	31 (20)	33 (20)
Previous symptoms while swimming in open water			
Yes	41 (31)	44 (29)	48 (29)
No	86 (65)	104 (68)	111 (67)
Had never swum in open water	5 (4)	4 (3)	6 (4)

Continuous data are presented as mean (SD) or median (IQR). Categorical data are presented as No. (%). IQR = interquartile range; MMU = mobile medical unit; PEP = positive expiratory pressure; SIPE = swimming-induced pulmonary edema.

a Missing data (n = 2).

b Missing data (n = 3).

c Missing data (n = 5).

d Missing data (n = 1).

e The question referred to the same year as the race.

### Symptom Duration

**Figure 2:**
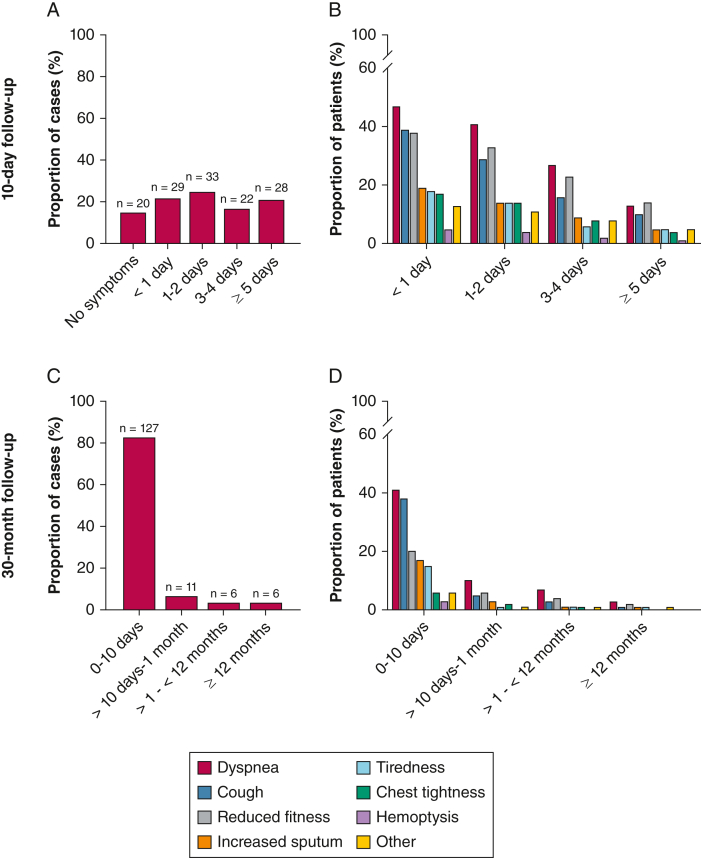
A-D, Symptom duration and reported symptoms after discharge from the mobile medical unit at 10-day follow-up (A and B) (n = 132) and 30-month follow-up (C and D) (n = 152). Data are presented as proportion of total number of SIPE cases. Multiple symptoms could be reported. For most of the cases, “other symptoms” referred to difficulties experienced during physical activity. SIPE = swimming-induced pulmonary edema.

The most common acute respiratory symptoms at the MMU were dyspnea and cough, but some also reported increased sputum and hemoptysis (Table 2). At the 10-day follow-up, 112 (85%) of the 132 cases reported symptoms that persisted after leaving the MMU. Of these, the vast majority described no asymptomatic interval (n = 106; 95%). Symptoms lasted longer than 2 days for 50 cases (38%), and 28 cases (21%) still had symptoms after 5 days (Fig 2A). Adjusted for age and hypertension, asthma was associated with a symptom duration of > 2 days (OR, 2.56; *P* = .045) (Table 3).

**Table 3:** Factors Associated With Symptom Duration > 2 Days

Variable	Unadjusted Analysis		Adjusted Analysis^a^	
	OR (95% CI)	*P* Value	OR (95% CI)	*P* Value
Age	0.98 (0.95-1.01)	.313	0.98 (0.94-1.02)	.276
Hypertension	1.26 (0.41-3.88)	.685	1.46 (0.46-4.63)	.522
Asthma	2.53 (1.01-6.32)	.047	2.56 (1.02-6.43)	.045

Logistic regression analysis for symptom duration > 2 days in patients included in the 10-day follow-up (n = 132).

a Overall percentage correct, 64.4%; Nagelkerke, 0.055.

Of 152 patients at the 30-month follow-up, 23 patients (15%) reported symptoms that persisted for more than 10 days, and 6 patients (4%) reported continuing symptoms even after 12 months (Fig 2C). Dyspnea, cough, and reduced fitness were the most common continuing symptoms mentioned in both follow-up interviews (Figs 2B, 2D).

### Recurrence of SIPE

At 30 months, 64 of 152 patients had swum in open water again after the swimming race. Of these, 18 patients (28%) reported episodes of respiratory symptoms while swimming in open water. Most of these patients reported respiratory symptoms on one or two occasions (15 patients) whereas two patients reported symptoms on three to five occasions and one patient more than 10 times. Three patients reported cough/dyspnea while swimming in a swimming pool, as did three additional patients who had not swum in open water again after the race. Of note, only three patients had lung ultrasound done during the recurrent episodes, all of which confirmed pulmonary edema. Adjusted for age and hypertension, asthma was associated with recurrence of SIPE at the 30-month follow-up (OR, 6.91; *P* = .022) (Table 4). In patients with recurrence of SIPE, 28% (5 of 18) had asthma, compared with 7% of patients (3 of 46) without recurrence.

**Table 4:** Factors Associated With Recurrence of SIPE

Variable	Unadjusted Analysis		Adjusted Analysis^a^	
	OR (95% CI)	*P* Value	OR (95% CI)	*P* Value
Age	0.97 (0.92-1.02)	.264	0.96 (0.91-1.02)	.171
Hypertension	0.84 (0.08-8.68)	.886	0.79 (0.06-10.08)	.853
Asthma	5.51 (1.16-26.24)	.032	6.91 (1.32-36.06)	.022

Logistic regression analysis for self-reported recurrence of SIPE in patients who reported they had swum in open water again in 30-month follow-up (n = 64). SIPE = swimming-induced pulmonary edema.

a Overall percentage correct, 73.4%; Nagelkerke, 0.148.

Of all 165 patients who received a diagnosis of SIPE, 160 had swum in open water either before or after the swimming race in Vansbro. Of these, 59 patients (37%) reported respiratory symptoms while swimming in open water on one or more occasions in addition to the current swimming race.

### Medical Evaluation During Follow-Ups

After observation or treatment at the MMU, 150 of 165 patients with SIPE could be discharged. However, 15 patients (9%) were either directly transferred to hospital (n = 13) or were recommended to have further medical assessment (n = 2) based on hypoxemia and/or respiratory symptoms. Twelve patients (7%) were admitted for inpatient care, and of these, four patients were admitted to the ICU. The number of days at hospital varied from 1 to 3 days. Most patients were discharged with the diagnosis “pulmonary edema” (n = 7) or “dyspnea” (n = 1). Three patients received a diagnosis of myocardial infarction based on ECG changes and troponin dynamics. However, all three subsequently underwent coronary angiography, with normal results. One patient had persistent symptoms of dyspnea after discharge and later received a diagnosis of bilateral pulmonary embolism.

In addition, 23 of 132 patients (17%) sought outpatient care within 10 days of the swimming race, for persistent respiratory symptoms and/or for a check-up. Of these, none were admitted for inpatient care. However, 13 patients underwent chest radiography, all with normal results.

Of 152 patients interviewed at 30 months, 7 (5%) received a diagnosis of asthma, 12 (8%) of hypertension, and 8 (5%) of heart disease after the swimming race. Four of the patients who developed heart disease were already mentioned above—three with myocardial infarction and one with pulmonary embolism. In addition, two patients received a diagnosis of myocardial infarction during the follow-up period and underwent percutaneous coronary intervention, one patient reported perimyocarditis, and one reported a finding of a mild enlargement of the ascending aorta on echocardiography.

### Long-Term Effects on General Health and Physical Activity Level

**Figure 3:**
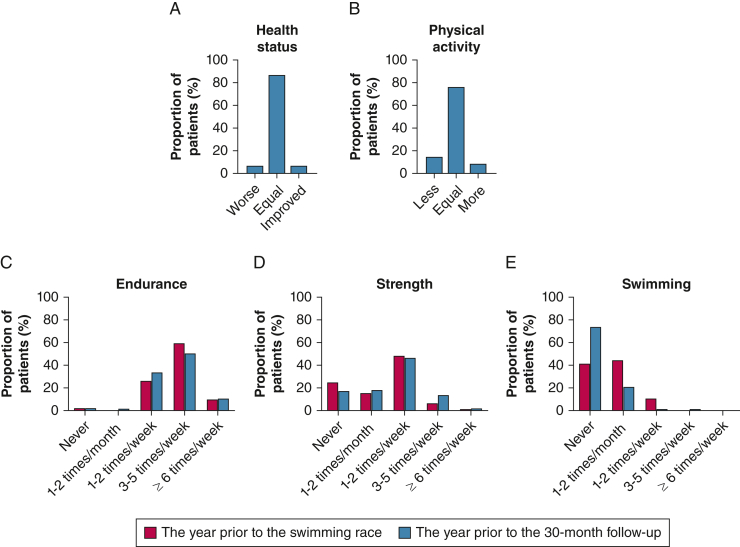
Long-term effects of SIPE on self-assessed general health, self-assessed physical activity level, and frequency of physical activity. A and B, Changes in general health status (A) and physical activity level (B) at 30 months compared with before experiencing SIPE (n = 152). C-E, Frequency of endurance (C), strength (D), and swimming exercise (E), the year before the swimming race and the year before the 30-month follow-up (n = 124). Only patients included in 2018 and 2019 were asked about their swimming exercise (n = 90). Data are presented as proportion of patients with SIPE. SIPE = swimming-induced pulmonary edema.

At the 30-month follow-up, most patients reported that their self-assessed general health (90%) and physical activity level (69%) had not been affected by experiencing SIPE. When rating their subjective health status compared with before SIPE, 93% reported equal or improved self-assessed general health, and 85% reported an equal or higher physical activity level (Figs 3A, 3B). The estimated frequency of endurance and strength exercise over the last year were comparable at both follow-ups, whereas the frequency of regular swimming exercise had decreased (Figs 3C-3E). The frequency of endurance and strength exercise was unaffected (*P* = 1.0 and .089, respectively) when comparing the proportions of patients answering that they “never” exercise and those answering “ever” (eg, at least once per month) at the two different follow-ups. However, the frequency of swimming exercise was lower the year before the 30-month follow-up compared with the year before the swimming race (*P* < .001).

Of 152 patients interviewed at 30 months, 88 patients (58%) reported that they had never swum in open water again after receiving a diagnosis of SIPE.

## Discussion

We present follow-up data up to 30 months for a large cohort of patients with SIPE. For a considerable portion of patients, respiratory symptoms persisted for longer than 2 days (38%) and even up to 5 days or longer (21%). Recurrence of respiratory symptoms during open water swimming occurred in 28% of participants over the 30-month follow-up period. Self-assessed general health and physical activity level were not affected by experiencing SIPE. However, more than one-half of the patients had not swum in open water again.

Previous data on time to recover from SIPE are inconsistent. Most of the reports describe a resolution of SIPE within 24 to 48 h.^7^^,^^13^^,^^14^^,^^18^, ^19^, ^20^, ^21^, ^22^, ^23^, ^24^, ^25^, ^26^, ^27^, ^28^ This timeframe has occasionally been used to confirm SIPE diagnosis and has even been used as a diagnostic criterion.^13^^,^^14^^,^^21^^,^^26^^,^^29^ However, some have observed symptoms and/or time to recovery for longer than 48 h, even up to 10 days.^19^^,^^22^^,^^30^, ^31^, ^32^, ^33^ Different definitions of “time to resolution” can account for some of the reported discrepancy in time to recovery. “Time to resolution” is sometimes based on symptom duration, the time to resolution of radiology-determined pulmonary edema, the time to discharge from hospital, or the time to resumed physical activity.^7^^,^^13^^,^^14^ Spirometry after an acute episode of SIPE has shown restrictive lung function impairment with reduction in FVC and FEV_1_ up to 12 h, with the reduction in FEV_1_ persisting up to 1 week after SIPE.^7^ These findings might reflect unresolved SIPE, which would be consistent with our report of symptoms lasting longer than 2 days. In the present study, 13 patients with continuing symptoms or need for medical check-up within 10 days showed normal chest radiography results, suggesting that symptoms of SIPE can persist after resolution of pulmonary edema on chest radiography. The large number of patients in this study who had symptoms for more than 2 days suggests that the 24 to 48-h timeframe for SIPE recovery needs to be reconsidered.

Within 30 months, 28% of these patients with SIPE reported recurrent episodes of respiratory symptoms while swimming in open water. These incidents most likely represented repeated episodes of SIPE. Several patients reported that they had never swum in open water again after experiencing SIPE, which might underestimate the risk of recurrence. This was supported by the fact that altogether 37% of participants reported recurrent respiratory symptoms while open water swimming, when also considering experiences before the race in Vansbro. Even though this cohort—which consists predominantly of middle-aged women—differs from most previous study groups of well-trained athletes, our recurrence rate is within the same range of previous findings.^7^, ^8^, ^9^, ^10^^,^^14^ The recurrence rate of SIPE reported in the literature is between 9% and 40% and varies on the basis of study population, the intensity and number of exposures, the length of the study period, and SIPE diagnosis criteria.^7^, ^8^, ^9^, ^10^^,^^13^^,^^14^ The vast majority of SIPE incidents occur during open water swimming, which suggests that a lower water temperature is associated with SIPE.^34^ However, in accordance with a previous study of triathletes, respiratory symptoms were also occasionally reported when swimming in a swimming pool in our study.^35^ Altogether, the relatively high risk of recurrence of SIPE suggests an individual predisposition to SIPE.

Preexisting cardiovascular comorbidities have been discussed as risk factors for immersion pulmonary edema in divers.^36^^,^^37^ In deaths among triathletes during swimming, left ventricular hypertrophy was overrepresented.^38^ In our relatively healthy group of patients with SIPE, consisting predominantly of middle-aged women, a lower prevalence of hypertension (11%) was found compared with the middle-aged Swedish population (22%).^39^ In contrast, we observed a higher prevalence of asthma (17%) compared with what has been reported for the middle-aged Swedish population (7%-11%).^40^^,^^41^ Interestingly, asthma was independently associated with both symptom duration > 2 days and recurrent SIPE symptoms after encountering SIPE. We speculate that SIPE and concurrent asthma may result in a worsening of symptoms and a corresponding extension of symptom duration. Similarly, a recurrent episode of SIPE may trigger an asthma exacerbation in patients with asthma. There is currently no evidence of a pathophysiologic connection between asthma or bronchial hyperreactivity and the development of SIPE. Also, most patients with recurrent SIPE had no previously known comorbid condition. Without a control group of swimmers without SIPE, the hypothesis of preexisting asthma as a risk factor for SIPE could not be tested in the present study, but merits consideration for future studies.

Most patients with SIPE who required acute hospital care had no other immediate cause for pulmonary edema than exposure to cold water swimming. Three patients received a diagnosis of myocardial infarction in adjacent to the race, which theoretically could have been the primary cause, or a consequence, of SIPE. However, all three patients had normal coronary angiography results, which is more indicative of a secondary myocardial strain. None of these patients reported having heart disease before the swimming race (data not shown). During the follow-up period of 30 months, 5%, 8%, and 5% of the patients reported receiving a diagnosis of either asthma, hypertension, or heart disease, respectively. Hypertension has been reported as a comorbid condition both in SIPE and immersion pulmonary edema among divers,^35^^,^^42^ and an increased incidence of hypertension over time has also been reported in this group.^1^ Some of the cases of hypertension diagnosed during the follow-up period might have been due to unknown or untreated high BP at the time of the swimming race. Because of the absence of a relevant control group in the present study, we could not evaluate the relative importance of individual cardiovascular and pulmonary risk factors and long-term consequences of SIPE.

Experiencing SIPE did not affect self-assessed general health and physical activity level up to 30 months for a majority of patients, except for a reduced frequency of swimming exercise. There are several potential explanations as to why swimming was less frequent at follow-up compared with the year before the swimming race in this group. In our experience, recreational swimmers usually practice indoor swimming before the *Vansbrosimningen* race, but do not otherwise swim on a regular basis. Since community-owned swimming pools in Sweden were temporarily closed in 2020-2021 due to the COVID-19 pandemic, access to indoor swimming was limited for the 2018 and 2019 cohorts. Because of the SIPE experience, there might also have been a general avoidance of swimming in this group, not only of swimming in open water.

A major strength of this study is the large original cohort of *Vansbrosimningen* including swimmers of all ages, both sexes, and with various swimming skills, which increases the generalizability of the data.^11^ The present data are unique because of the large number of SIPE cases followed for up to 30 months. Further strengths are the low number of participants lost to follow-up and the fact that the questionnaires were completed as telephone interviews, with the opportunity to clarify questions and answers. However, even with a large number of SIPE cases compared with previous literature on this topic, absolute numbers were still small, and the outcome of multivariable regression must be considered as hypothesis generating. Another potential limitation was the shift in diagnostic criteria over the study period. However, with more knowledge at the time of the 30-month interview, we concluded that the use of objective findings of pulmonary edema on LUS or the validated algorithm was more reasonable than the use of primary diagnostic criteria (Table 2). Recurrent episodes of respiratory symptoms while swimming were not verified by clinical examinations or radiology. It is, however, reasonable that these symptoms were caused by SIPE, as the patients could link their symptoms to a prior, documented SIPE episode. In follow-up interviews, recall and self-reporting bias must be considered. With this said, we tried to verify symptoms and discuss alternative diagnoses with the patients. The lack of a thorough validation of the questionnaires can present another limitation of the study even if we did a content validity process.

## Interpretation

The present large cohort study challenges the established hallmark of SIPE duration of < 48 h, whereas the recurrence of SIPE was within the previously reported range. Up to the 30-month follow-up after SIPE, self-assessed general health and physical activity level were not affected by experiencing SIPE. These results provide valuable information on short- and long-term outcomes for swimmers, swim event organizers, and health care professionals.

## Funding/Support

Financial support was provided by the Center for Research and Development, 10.13039/501100007051Uppsala University/Region Gävleborg and the Center for Clinical Research Dalarna-Uppsala University.

## Financial/Nonfinancial Disclosures

None declared.

## Supplementary Data

e-Online Data

e-Online Data

e-Online Data
