# Supplementary material for: Symptom Duration, Recurrence, and Long-Term Effects of Swimming-Induced Pulmonary Edema: A 30-Month Follow-Up Study
Source: Chest. 2023 Jul 5;164(5):1257–67. doi: 10.1016/j.chest.2023.06.041 (PMC10635841; doi:10.1016/j.chest.2023.06.041)
Supplement: e-Online Data [file mmc1.docx]

**Supplemental material**

Symptom duration, recurrence and long-term effects of swimming-induced pulmonary edema – a 30-month follow-up study

Linda Kristiansson, Claudia Seiler, Daniel Lundeqvist, Annika Braman Eriksson, Josefin Sundh, Maria Hårdstedt.

**E-figure legends**

E-figure 1—Algorithm used for SIPE diagnosis in patients with acute onset of cough and/or dyspnea during swimming in open water and no LUS. The algorithm is based on peripheral oxygen saturation and crackles on lung auscultation, with excessive sputum and/or hemoptysis supporting diagnosis. Patients with findings of crackles and peripheral oxygen saturation ≤95% could be diagnosed with SIPE based on clinical evaluation. For patients with findings of crackles or peripheral oxygen saturation ≤95%, LUS is suggested for differential diagnosis if available. Previously published by Hårdstedt et al, CHEST 2020 and 2021. LUS = lung ultrasound. SIPE = swimming-induced pulmonary edema.

E-figure 2—Translated questions used at MMU, 10-day and 30-month follow-up. Interviews were conducted on site at MMU and by phone for 10-day and 30-month follow-up. COPD = Chronic obstructive pulmonary disease. MMU = Mobile medical unit. SIPE = swimming-induced pulmonary edema.
