# Supplementary material for: Symptom Duration, Recurrence, and Long-Term Effects of Swimming-Induced Pulmonary Edema: A 30-Month Follow-Up Study
Source: Chest. 2023 Jul 5;164(5):1257–67. doi: 10.1016/j.chest.2023.06.041 (PMC10635841; doi:10.1016/j.chest.2023.06.041)
Supplement: e-Online Data [file mmc2.docx]

**Supplemental material**

Symptom duration, recurrence and long-term effects of swimming-induced pulmonary edema – a 30-month follow-up study

Linda Kristiansson, Claudia Seiler, Daniel Lundeqvist, Annika Braman Eriksson, Josefin Sundh, Maria Hårdstedt.

**E-figure 2 — Translated questions used at MMU, 10-day and 30-month follow-up**

**MMU (at the race):**

Respiratory symptoms: □ Cough

□ Dyspnea

□ Increased amount of mucus

□ Blood-tinged sputum

Daily smoker during any period in the last year?

□ Yes □ No

Have you previously experienced breathing problems or coughing when swimming in open water? □ Yes □ No □ I have never svum in open water

How many times have you practiced swimming in open water this year? □ Never before □ 1-2 times before the race

□ 3-5 times before the race □ More than 5 times before the race

Previously known respiratory disease? □ Yes

□ No If yes: □ Asthma

□ COPD

□ Other pulmonary disease

Previously known heart disease? □ Yes □ No If yes: □ Percutaneous coronary intervention with stent/myocardial infarction

□ Arrhythmia

□ Pericarditis/myocarditis

□ Other heart disease

Previously known high blood pressure? □ Yes

□ No

Other disease? □ Yes

□ No If yes: which?____________

Current medication? □ Yes

□ No

If yes: which?____________

**10-day follow-up (E2):**

Have you experienced any symptoms since you left the health care unit in Vansbro?

□ No, no symptoms

□ Yes, I have had symptoms: □ Cough

□ Breathing problems

□ Increased amount of mucus in the airways/wheezy

□ Blood-tinged/dark mucus from the respiratory tract

□ Reduced fitness

□ Other, what? ____________________

For how long did you experience symptoms after the race?

□ I have been feeling well since I left the health care unit in Vansbro

□ <1 day

□ 1-2 days

□ 3-4 days

□ ≥ 5 days

Are you completely symptom free now? □ Yes

□ No

If not, which symptoms?

□ Cough

□ Breathing problems

□ Increased amount of mucus in the airways/wheezy

□ Blood-tinged/dark mucus from the respiratory tract

□ General reduced fitness

□ Other, what? _____________________

Did the symptoms first resolve and then return? (i.e. did you experience a free interval regarding symptoms) □ No

□ Yes If yes, for how long time did you have no symptoms? ___ If yes, when did the symptoms return? _______________

Have you sought medical attention after you left health care in Vansbro? □ No

□ Yes If yes, for what did you sought medical attention? _____

If yes, where did you sought medical attention? _______ If yes, when did you sought medical attention? ________ If yes, which investigations were performed and what did they show? _______

If the reason you sought medical attention was related to pulmonary edema (SIPE, e.g. heart/lung problems), may we then have access to your medical record? ______

How often did you perform endurance exercise in the last 12 months? Intensity about "moderate", sweaty (type of exercise running, Nordic walking, cycling, cross trainer, swimming, etc.) Duration approx. 20min □ Never

□ 1-2 times/month

□ 1-2 times/week

□ 3-5 times/week

□ 6-7 times/week

How often did you perform strength exercise in the last 12 months?

□ Never

□ 1-2 times/month

□ 1-2 times/week

□ 3-5 times/week

□ 6-7 times/week

How often did you practice swimming (in a pool or in open water)?

During the two previous months? During the twelve previous months?

□ Never □ Never

□ 1-2 times/month □ 1-2 times/month

□ 1-2 times/week □ 1-2 times/week

□ 3-5 times/week □ 3-5 times/week

□ 6-7 times/week □ 6-7 times/week

**30-month follow-up (E3):**

For how long time after the race did you experience symptoms related to pulmonary edema (SIPE)? □ ≤ 10 days

□ > 10 days to < 1 month

□ > 1 month to < 12 months

□ ≥ 12 months

□ Don’t remember

If yes, which symptoms?

□ Cough

□ Breathing problems

□ Increased amount of mucus in the airways/wheezy

□ Blood-tinged/dark mucus from the respiratory tract

□ General reduced fitness

□ Other, what? __________________________________

□ Don’t remember

How many times have you swum in open water after you experienced pulmonary edema (SIPE) at Vansbrosimningen in 20XX? □ Never

□ 1-2 times

□ 3-5 times

□ 6-10 times

□ >10 times

Have you experienced respiratory problems or coughing when swimming in open water after you had pulmonary edema (SIPE) at Vansbrosimningen in 20XX?

□ Yes

□ No

□ Not swum in open water

If yes, which symptoms?

□ Cough

□ Respiratory problems

□ Increased amount of mucus in the airways/wheezy

□ Blood-tinged/dark mucus from the respiratory tract

□ General reduced fitness

□ Other, what? __________________________________ □ Don’t remember

If yes, how many times?

□ 1-2 times

□ 3-5 times

□ 6-10 times

□ >10 times

If yes, describe? (when, where, how?) ____________________________

(lake, river, water temperature, exercise/competition, surrounding factors e.g. stress)

Have you sought medical attention or gone through any medical investigations since you experienced pulmonary edema (SIPE)?

□ No

□ Yes If yes, for what did you sought medical attention? ______

If yes, where did you sought medical attention? _______ If yes, when did you sought medical attention? ________ If yes, which investigations were performed and what did they show? ________________________ If the reason you sought medical attention was related to pulmonary edema (SIPE, e.g. heart/lung problems), may we then have access to your medical record? □ Yes

The patient sends medical record to us: □ Yes

Double-check and confirm the medical diagnosis in questionnaire E1:

Diagnosis asthma by doctor □ Yes □ No

Diagnosis heart disease by doctor □ Yes □ No

Diagnosis high blood pressure by doctor □ Yes □ No

Have you, since you were diagnosed with pulmonary edema (SIPE) in 20XX, received any of the following diagnosis by a doctor? Asthma □ Yes □ No

Chronic obstructive pulmonary disease □ Yes □ No

Other pulmonary disease □ Yes □ No

If yes, which one______________

Heart disease □ Yes □ No

If yes, □ Percutaneous coronary intervention with stent/myocardial infarction

□ Arrhythmia (heart rhythm disorder)

□ Pericarditis/myocarditis (inflammation in heart sac/muscle)

□ Other heart disease

If yes, which one______________

High blood pressure □ Yes □ No Other disease □ Yes □ No

If yes, which one______________

Do you use any medications?

□ Yes □ No

If yes, which one______________

Do you have medication prescribed for asthma? □ Yes □ No

Do you have medication prescribed for high blood pressure?

□ Yes □ No

How often did you perform endurance exercise in the last 12 months? Intensity about “moderate", sweaty (type running, Nordic walking, cycling, cross trainer, swimming, etc.) Duration approx. 20min

□ Never

□ 1-2 times/month

□ 1-2 times/week

□ 3-5 times/week

□ 6-7 times/week

How often did you perform strength exercise in the last 12 months?

□ Never

□ 1-2 times/month

□ 1-2 times/week

□ 3-5 times/week

□ 6-7 times/week

How often did you practice swimming (in a pool or in open water)?

During the two previous months? During the twelve previous months?

□ Never □ Never

□ 1-2 times/month □ 1-2 times/month

□ 1-2 times/week □ 1-2 times/week

□ 3-5 times/week □ 3-5 times/week

□ 6-7 times/week □ 6-7 times/week

Has experiencing pulmonary edema (SIPE) affected your regular exercise?

□ Yes

□ No

If yes, in which way? ________ If yes, which category of exercise has been affected? _______

E.g.: exercise less, exercise differently, avoids swimming, do not exercise in open water.

I exercise after experiencing pulmonary edema (SIPE):

□ Much less

□ Less

□ Equal

□ More

□ Much more

Has experiencing pulmonary edema (SIPE) affected your general health? □ Yes

□ No

If yes, in which way? ________

I would describe my general health status after experiencing pulmonary edema (SIPE) as:

□ Much worse

□ Worse

□ Equal

□ Improved

□ Much better
