# Supplementary material for: Symptom Duration, Recurrence, and Long-Term Effects of Swimming-Induced Pulmonary Edema: A 30-Month Follow-Up Study
Source: Chest. 2023 Jul 5;164(5):1257–67. doi: 10.1016/j.chest.2023.06.041 (PMC10635841; doi:10.1016/j.chest.2023.06.041)
Supplement: e-Online Data [file mmc3.docx]

| **E-Table 1. The different cohorts used to evaluate outcome measures** | | | | | | |
| --- | --- | --- | --- | --- | --- | --- |
|  | **SIPE cases at the MMU** | **10-day follow-up** | **30-month follow-up** | **Follow-up for 10 days and 30 months** | |  |
| **Recurrence of SIPE** |  |  |  |  | | |
| **Acute medical care** | n =165 |  |  | |  | |
|  |  |  |  | |  | |
| **Symptom duration <10 days**  **Medical assessment <10 days** |  | n =132 |  | |  | |
| **Symptom duration >10 days**  **Recurrence of SIPE**  **Medical evaluation during follow-up**  **Self-assessed general health and**  **physical activity level** |  |  | n =152 | |  | |
| **Compared frequency of physical activities**  **between follow-ups (different modalities)** |  |  |  | | n =124 | |
| MMU = mobile medical unit, SIPE = swimming-induced pulmonary edema. | | | | | | |

Supplemental material

Symptom duration, recurrence and long-term effects of swimming-induced pulmonary edema – a 30-month follow-up study

Linda Kristiansson, Claudia Seiler, Daniel Lundeqvist, Annika Braman Eriksson, Josefin Sundh, Maria Hårdstedt.
